# CD9+ and CD82+ extracellular vesicles in synovial fluid differentiate aseptic from septic endoprosthesis loosening

**DOI:** 10.20517/evcna.2025.11

**Published:** 2025-07-10

**Authors:** Tobias Tertel, Vera Rebmann, Charlotte Bielefeld, Marcel Haversath, Marcus Jäger, Alexander Wegner, André Busch, Bernd Giebel

**Affiliations:** ^1^Institute for Transfusion Medicine, University Hospital Essen, University of Duisburg-Essen, Essen 45147, Germany.; ^2^Department of Orthopedics and Trauma Surgery, University of Duisburg-Essen, Essen 45147, Germany.; ^3^Institute of Pathology Nordhessen, Kassel 34119, Germany.; ^4^Department of Trauma surgery, Orthopedics and Hand surgery, Klinikum Wolfsburg, Wolfsburg 38440, Germany.; ^5^Katholisches Klinikum Essen Philippus-Stift, Essen 45355, Germany.

**Keywords:** Extracellular vesicles, synovial fluid, imaging flow cytometry, biomarkers, endoprosthesis

## Abstract

**Aim:** Extracellular vesicles (EVs) hold great promise as emerging biomarkers for a variety of diseases. However, their clinical application is still hindered by complex and time-consuming isolation procedures. A clinically relevant scenario where improved biomarker-based diagnostics are urgently needed is prosthetic joint loosening, which may result from either aseptic inflammation or periprosthetic joint infection (PJI) - two conditions requiring fundamentally different therapeutic approaches. This study investigated whether imaging flow cytometry (IFCM) enables the discrimination between aseptic and septic loosening by profiling EVs directly in minimally processed synovial fluid.

**Methods:** We analyzed synovial fluid from 35 aseptic and 13 septic cases using IFCM to detect surface marker-defined EV subpopulations without prior isolation. Samples were classified based on clinical and microbiological findings. Marker abundance was quantified and analyzed using logistic regression.

**Results:** Septic loosening was associated with significantly increased levels of CD82^+^ EVs and decreased levels of CD9^+^ EVs. CD82^+^ EVs showed a sensitivity of 83.3% and specificity of 90.0%, while CD9^+^ EVs demonstrated 100% specificity but lower sensitivity (58.8%) for aseptic loosening. CD82^+^ EV abundance was identified as an independent predictor of septic loosening.

**Conclusion:** IFCM enables rapid and direct detection of diagnostically relevant EVs in native synovial fluid. CD9^+^ and CD82^+^ EVs serve as promising biomarkers for distinguishing aseptic from septic endoprosthesis loosening, offering a fast and robust diagnostic tool that may complement current clinical diagnostics and support timely treatment decisions.

## INTRODUCTION

Extracellular vesicles (EVs), including exosomes, are nanosized membrane-enclosed particles secreted by nearly all cell types^[1,2]^. They carry diverse molecular cargo, including proteins, RNAs, and lipids, that reflect the physiological or pathological state of their cells of origin. Due to these properties, EVs have emerged as promising biomarkers in various diseases. Their small size and heterogeneous composition, however, complicate their detection and analysis, particularly when using conventional bulk analysis methods.

In 2011, we and others introduced nanoparticle tracking analysis (NTA) as a single EV analysis platform^[3,4]^. However, over the years, it became obvious that many EV samples contain far more non-EV-related particles than EVs and that NTA and other particle quantification devices including resistive pulse sensing^[5]^ and dynamic light scattering^[6]^ in their traditional forms cannot discriminate EVs from other particles of similar sizes^[7]^. With the overall increasing interest in EVs, advanced methods have been introduced to the field that rely on the specific detection of labeled EVs, among them fluorescence NTA, advanced plasmon resonance^[8,9]^, sophisticated fluorescence microscopy^[10]^, and a new generation of flow cytometers being optimized for small particle analyses^[11]^. We and others have qualified imaging flow cytometry (IFCM) as a powerful platform for single-EV analysis^[12,13]^. However, labeling and analyses of exosome-sized or sEVs at the single EV level pose several challenges. These challenges and potential pitfalls have been comprehensively described in a recent collaborative publication by experts in the EV analysis field^[14]^. Furthermore, a framework has been created to standardize the analysis of EVs and to gain more transparency in EV analyses^[15]^.

Unlike most EV characterization methods, which require extensive purification steps, our IFCM-based workflow allows direct analysis of fluorescently labeled EVs in minimally processed biofluids. We have optimized this approach using antibody-based staining or novel membrane dyes, eliminating the need for laborious pre- or post-labeling steps^[16,17]^. In a previous study, we applied this strategy to sera from COVID-19 patients and identified EV signatures correlating with disease severity^[18]^. To our knowledge, IFCM is currently the only EV analysis platform with automated sample handling, enabled by the AMNIS ImageStream MX Mark II. In contrast to many approaches that require EV isolation or omics-level profiling, our method enables direct phenotypic quantification of surface marker-defined EV subpopulations in native synovial fluid. This reduces sample handling time and preserves physiological context, supporting its use for translational diagnostics. These advantages make IFCM-based EV phenotyping a practically feasible and translationally relevant approach for biomarker discovery in localized disease settings.

Building on these advantages, we extended our approach to a less commonly studied biofluid, synovial fluid, collected from patients undergoing revision arthroplasty due to septic or aseptic prosthesis loosening.

This investigation is based on a clinically relevant problem. Total joint arthroplasty (TJA), particularly in the hip and knee, is a widely accepted treatment for advanced osteoarthritis, with generally low complication rates^[19]^. Nevertheless, prosthetic failure remains a significant challenge. Aseptic loosening, often caused by wear debris-induced immune activation, leads to osteolysis and mechanical failure of the implant^[20,21]^. Conversely, periprosthetic joint infections (PJIs) also induce immune-driven osteolysis but require fundamentally different treatment strategies^[22]^.

The preoperative discrimination between aseptic failure and PJl is crucial because the treatment of aseptic complications fundamentally differs from the treatment of PJI^[23,24]^. In cases of PJI, particularly in chronic infections, revision surgery typically involves a two-stage procedure with the temporary placement of an antibiotic-impregnated cement spacer to eradicate the infection. Patients often experience immobilization between these two surgical stages. In contrast, aseptic complications can usually be addressed through a one-stage procedure^[25]^. The diagnosis of PJI remains challenging and time-consuming, with up to 20% of cases obtaining false negative results from microbiological diagnostics^[26]^. Therefore, various diagnostic criteria based on clinical, laboratory, microbiological, histological, and intraoperative findings have been established for the diagnosis of PJI^[27]^. In routine diagnostic, serum and synovial biomarkers such as C-reactive protein (CRP), alpha-1-defensin, and white cell counts are often used to indirectly diagnose PJI^[28-30]^. Nevertheless, until now, no "gold standard" diagnostic method has been established to rule out PJI^[31]^.

Therefore, our study aimed to explore the utility of EVs as a supplementary diagnostic tool for distinguishing between aseptic complications and PJI in a relevant patient cohort. To achieve this, we utilized an advanced antibody panel to analyze EV populations in the synovial fluid of patients experiencing either aseptic or septic endoprosthesis loosening through IFCM. This approach enabled us to identify distinct EV populations with varying abundances in the two patient groups.

## METHODS

After approval by the local institutional ethics committee (18-8042-BO), the patients for this prospective study were recruited from Department of Orthopedics and Trauma Surgery at the University of Duisburg-Essen, Germany. The study was performed in accordance with the Declaration of Helsinki. All individuals suffered from persisting pain^[32]^ after hip, knee, or shoulder arthroplasty. All patients signed informed consent forms prior to inclusion.

Inclusion criteria were enough synovial fluid gained pre- or intraoperatively for all analyses as well as full clinical and laboratory data. Patients were excluded if they showed signs of early postoperative PJI (8 weeks) due to reduced reliability of synovial and serologic markers shortly after surgery^[33,34]^. Inflammatory comorbidities (rheumatism, chronic bowel disorder), metallosis, and previous or concomitant antibiotic therapy were other criteria leading to exclusion.

### Patients

Before surgery, the patients' medical history, clinical examinations, and laboratory values, including CRP, were collected as part of routine preoperative diagnostic testing. Joint aspiration fluid was collected routinely under aseptic conditions, i.e., by using a threefold skin disinfection technique. Based on the results of these tests, the patients were classified as having either aseptic complications or PJI according to the Musculoskeletal Infection Society (MSIS) criteria^[35]^. To confirm the diagnosis of PJI, at least one major criterium or, alternatively, four out of six minor criteria need to be met [Supplementary Tables 1 and 2].

### Sex as a biological variable

Our study examined both male and female human subjects. The patient cohort comprised 22 women and 13 men with aseptic endoprosthesis loosening, and 7 women and 6 men with septic endoprosthesis loosening. There were no significant differences in sex, age, or BMI between the two groups, and similar findings were reported for both sexes. Therefore, the findings of this study are expected to be relevant for both male and female patients [Supplementary Table 2].

### Sample preparation

Synovial fluid was collected via joint puncture, avoiding an admixture of blood with an 18-gauge needle. Synovial fluid was aseptically aliquoted into sterile tubes and centrifuged for the first time at 2,000 × *g* for 8 min at 4 °C. The supernatant (SN) was kept on ice for less than 1 h until samples were further processed. For the removal of larger debris, they were centrifuged for 10 min at 4 °C with 10,000 × *g*. The SN was transferred into new containers. 10 µL aliquots were treated with either 0.25 µL of anti-human CD9 PE (EXBIO, Vestec, Czech Republic), anti-human CD63 APC (EXBIO), anti-human CD66b (LeukoCom GmbH, Essen, Deutschland), anti-human CD82 (BioLegend, San Diego, CA, USA), or anti-human HLA-DR (Beckman Coulter, Krefeld, Germany) antibodies diluted in 10 µL PBS and incubated at room temperature for 2 h. Thereafter, PBS was added to a final volume of 100 µL, and samples were directly analyzed by IFCM. In agreement with the MiFlowCyt-EV criteria^[15]^, in addition to buffer only and buffer plus antibody controls, controls with fluorochrome-conjugated isotype antibodies and detergent lysis with 1.25% NP-40 (solved in PBS) controls were prepared^[15]^.

All samples were analyzed in technical duplicates on the AMNIS ImageStreamX Mark II Flow Cytometer (AMNIS/Luminex, Seattle, WA, USA) using the built-in autosampler for U-bottom 96-well plates (Corning Falcon, cat 353077) and the IDEAS software 6.2. Acquisition times were 5 min per well at 60x magnification and a low flow rate (0.3795 ± 0.0003 μL/min, directly determined by the system) with the removed beads option deactivated as described previously^[12]^. The data were analyzed as described previously^[18]^. We calibrated the system using MESF (Molecules of Equivalent Soluble Fluorochrome) and determined the detection threshold for EVs in synovial fluid. Without prior enrichment, the detection limit for marker-positive EVs is approximately 10^5^ objects per mL of synovial fluid. Samples with lower concentrations would require enrichment, which introduces bias and can lead to EV loss during isolation. Detailed machine settings are provided in Supplementary Tables 3 and 4.

### Statistical analysis

The statistical analyses were performed using IBM SPSS Statistics 25 and Graph Pad Prism 8 software packages. After testing for Gaussian distribution, the differences in continuous data were assessed between the two groups by the Mann-Whitney test for non-parametric data. The median and range were reported for each marker. A receiver operating characteristic (ROC) analysis was performed (BIAS software program 11.08; https://www.bias-online.de/) to define optimal cut-off values concerning sensitivity and specificity to translate continuous patient characteristics into dichotomous variables that enable optimal separation of patients with aseptic and septic endoprosthesis loosening. Binomial logistic regression was used to analyze the influence of certain parameters on the prediction of aseptic or septic endoprosthesis loosening. A *P*-value less than 0.05 was considered statistically significant.

## RESULTS

During the recruitment period, the synovial fluid of a total of 35 patients fulfilling the MSIS criteria for aseptic joint effusion was included in this study [Supplementary Table 1]. The patient group comprised 22 women and 13 men (mean age 72.1 ± 12.9 years; range: 25-88). In this cohort, 15 knees, 19 hips, and 1 shoulder were replaced. The mean BMI was 30.0 ± 7.5 (range: 21.4-52.0) kg/m^2^ [Supplementary Table 2]. In addition, the synovial fluid of 13 patients fulfilling MSIS criteria for PJI was included [Supplementary Table 1]. The PJI group consisted of 7 women and 6 men with a mean age of 69 ± 15.6 (range: 35-89) years. In this group, 7 knees, 5 hips, and 1 shoulder were replaced. The mean BMI was 32.4 ± 9.3 (range: 20.2-50.9) kg/m^2^. Nine joint fluid aspirations from the latter group tested positive for microbiological culture. Bacteria were identified in 9 (69.2%) of the 13 patients. In 4 patients (30.8%) of this group, no bacteria could be isolated after 14 days of incubation. Of note, there were no significant differences in sex, age, and BMI between the two groups (*P* > 0.05) [Supplementary Table 2].

### IFCM allows EV analysis in cleared synovial fluid

To analyze EV populations in cleared synovial fluid of the recruited cohort, we aimed to use an extended antibody panel. Notably, many commercially available fluorochrome-conjugated antibodies, which are certified for cellular analyses, do not meet the prerequisites for single EV analyses. Either they contain EV-like objects themselves or do not label EVs sufficiently for detection. Thus, each antibody used for single-EV analyses had to be specifically qualified up front. In this study, we focused on antibodies recognizing antigens known to be expressed on different blood cell types, including thrombocytes. In detail, we used antibodies that we had qualified for the analysis of blood samples, similar to our previous study^[18]^. Specifically, we used antibodies against CD9, CD25, CD29, CD36, CD41, CD63, CD66b, CD71, CD82, CD107a, HLA-ABC, and HLA-DR.

**Figure 1:**
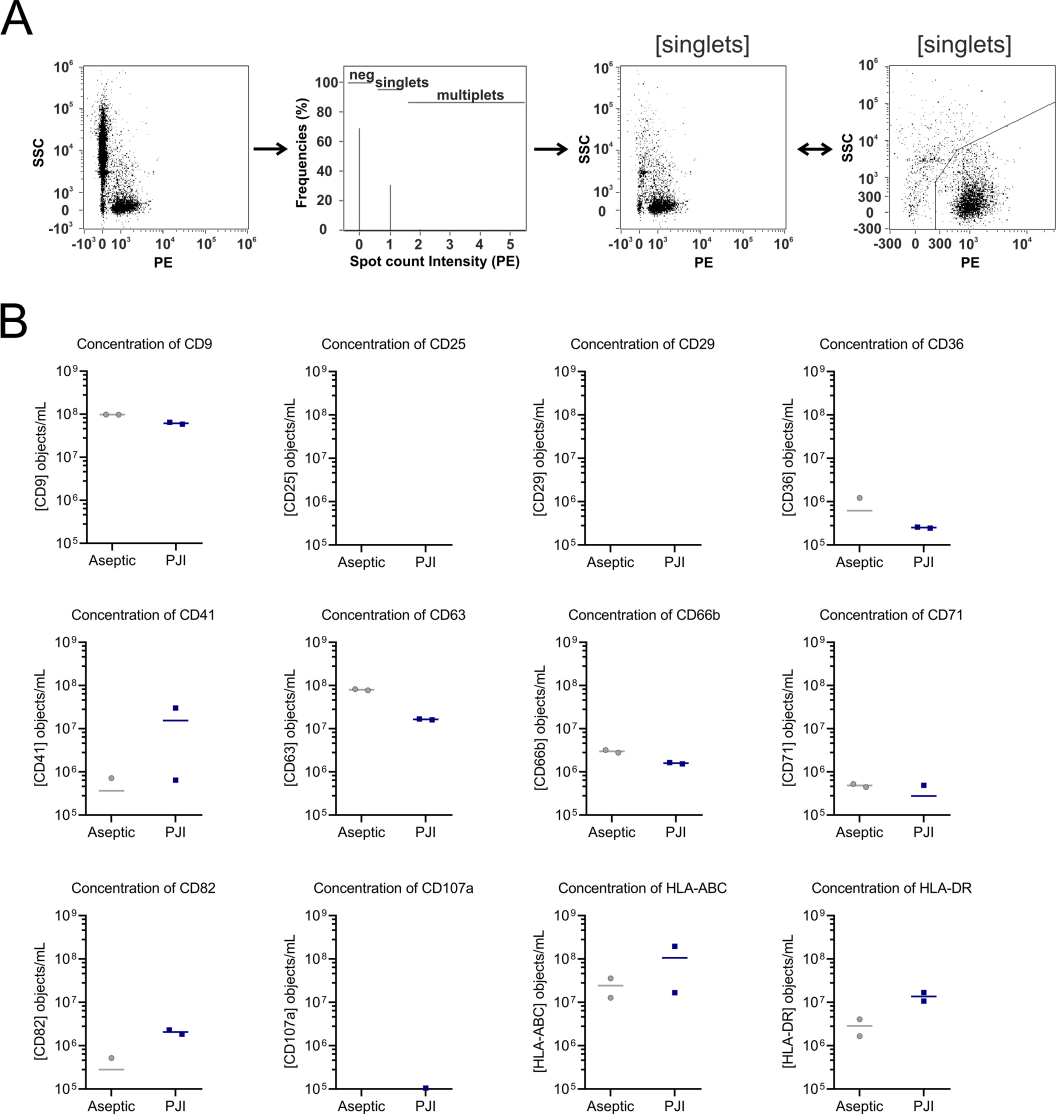
Analysis of EV populations in synovial fluid with an extended antibody panel. Following staining with different antibodies that had been qualified for the single EV analyses, synovial fluid aliquots were analyzed by imaging flow cytometry. (A) Applied gating strategy; events with coincidences (multiplets) were excluded from analyses using the Spot Count feature within the AMNIS Imagestream software. Plots show events before and after gating, including a zoom-in of the plot with the gated events (B) Concentration of the EVs recognized after labeling with each of the 12 antibodies used in this study. EV populations were recorded in either two samples of synovial fluid of patients with aseptic and septic endoprosthesis loosening, respectively. Drawing Software: CorelDraw (2021, Version 23.1.0.389). (A) plots exported from the software (IDEAS), with axis font sizes adjusted in CorelDraw to enhance visibility; (B) GraphPad. (A) and (B) compiled with CorelDraw. EV: Extracellular vesicle; PJI: periprosthetic joint infection.

In a preliminary experiment, we investigated the presence of EV populations in the synovial fluid of two patients with aseptic endoprosthesis loosening and two patients with PJI, using different antibodies. Samples with volumes greater than 200 µL were selected for these analyses, and each antibody was applied to a 10 µL aliquot of each of these samples. After incubation, samples were analyzed by IFCM using a gating strategy excluding coincidences, as illustrated in Figure 1. We deemed only EV populations with over 500 recorded objects as reliable, equivalent to more than 10^5^ objects per 1 mL of the original synovial fluid. Only EVs that exceed the detection threshold, based on our calibration, were included in the analysis. This threshold ensures that detected EVs are reliably characterized, minimizing background noise and non-specific particles. This approach contributes to the reproducibility of the measurements across samples. In all four samples stained with anti-CD25, anti-CD29, and anti-CD107a antibodies, we detected fewer than 500 objects per mL, which may reflect true biological absence or technical limitations such as insufficient antibody binding or low antigen density on the vesicles. Given the complexity of synovial fluid and the pilot nature of this study, we cannot definitively distinguish between these possibilities.

Considering the limiting volumes of the other patient samples, we streamlined the comparison among the remaining patient samples to five different antibodies. We intentionally chose the established EV markers CD9 and CD63, consistently showcasing similar object counts across every sample pair but revealing notable differences among them. Additionally, we included CD82, another tetraspanin, notably showing higher abundances in the PJI sample pair. Our choice of CD66b, a neutrophil marker, also revealed consistent object numbers across each sample pair and hinted at potential contrasts among them. Finally, HLA-DR, similar to CD82, displayed markedly higher abundances in the PJI sample pair, completing our panel of markers for comprehensive analysis.

### Single EVs can be reliably labeled with antibodies in marginally processed synovial fluid

**Figure 2:**
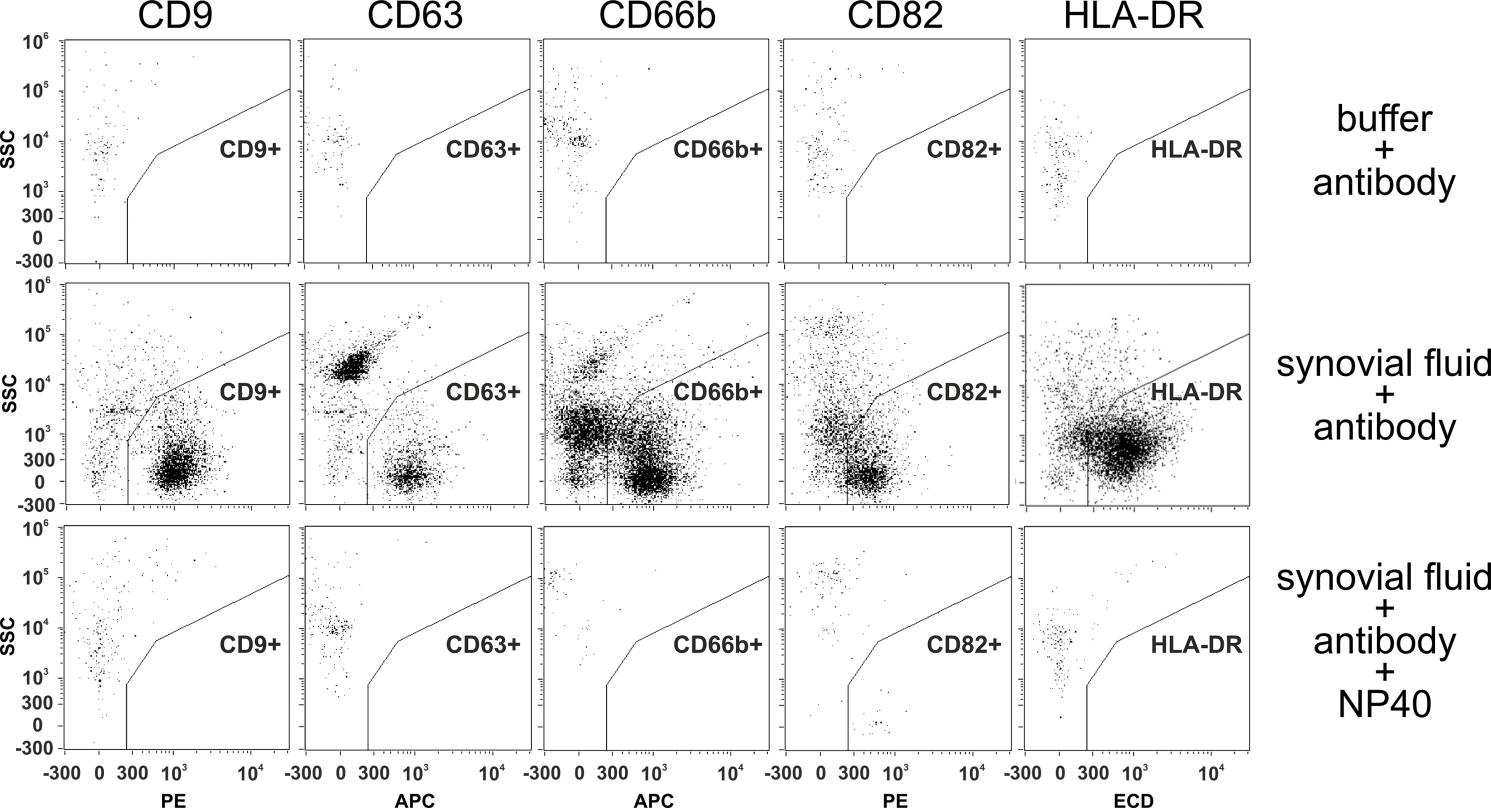
The selected anti-CD9, anti-CD63, anti-CD66b, anti-CD82, and anti-HLA-DR antibodies enable labeling of detergent-sensitive objects in marginally processed synovial fluid. For the evaluation of background signals, antibodies were applied to PBS (1st row); for the detection of EVs, aliquots of a synovial fluid sample that had been preprocessed by 2,000 and 10,000 × *g* centrifugation were labeled with each of the five different antibodies (2nd row). To approve the EV nature of identifiable objects, detergent controls were performed; specifically, NP40 was added to labeled synovial samples before analysis (3rd row). Samples were analyzed by imaging flow cytometry using the gating strategy as depicted in Figure 1A. Drawing Software: CorelDraw. EV: Extracellular vesicle.

Before exploring the EV populations in marginally processed synovial fluids of all collected patients, we performed controls as recommended by the MIFlowCyt-EV consortium on aliquots of a given larger volume synovial fluid sample^[15]^. To this end, controls with all reagents but without synovial fluid, i.e., PBS and any of the 5 different selected antibodies, and lysis controls were performed, in which NP40 was provided to regularly stained synovial fluid samples. As indicated in Figure 2, specific EV populations were recovered after the normal labeling, but not in the two-control series. Thus, the labeling procedure and the usability of the five selected antibodies were approved for the labeling of single EVs in marginally processed synovial fluids.

### The synovial fluids of patients with aseptic endoprosthesis loosening contained significantly higher CD9^+^ and lower CD82^+^ EV levels than PJI patients

**Figure 3:**
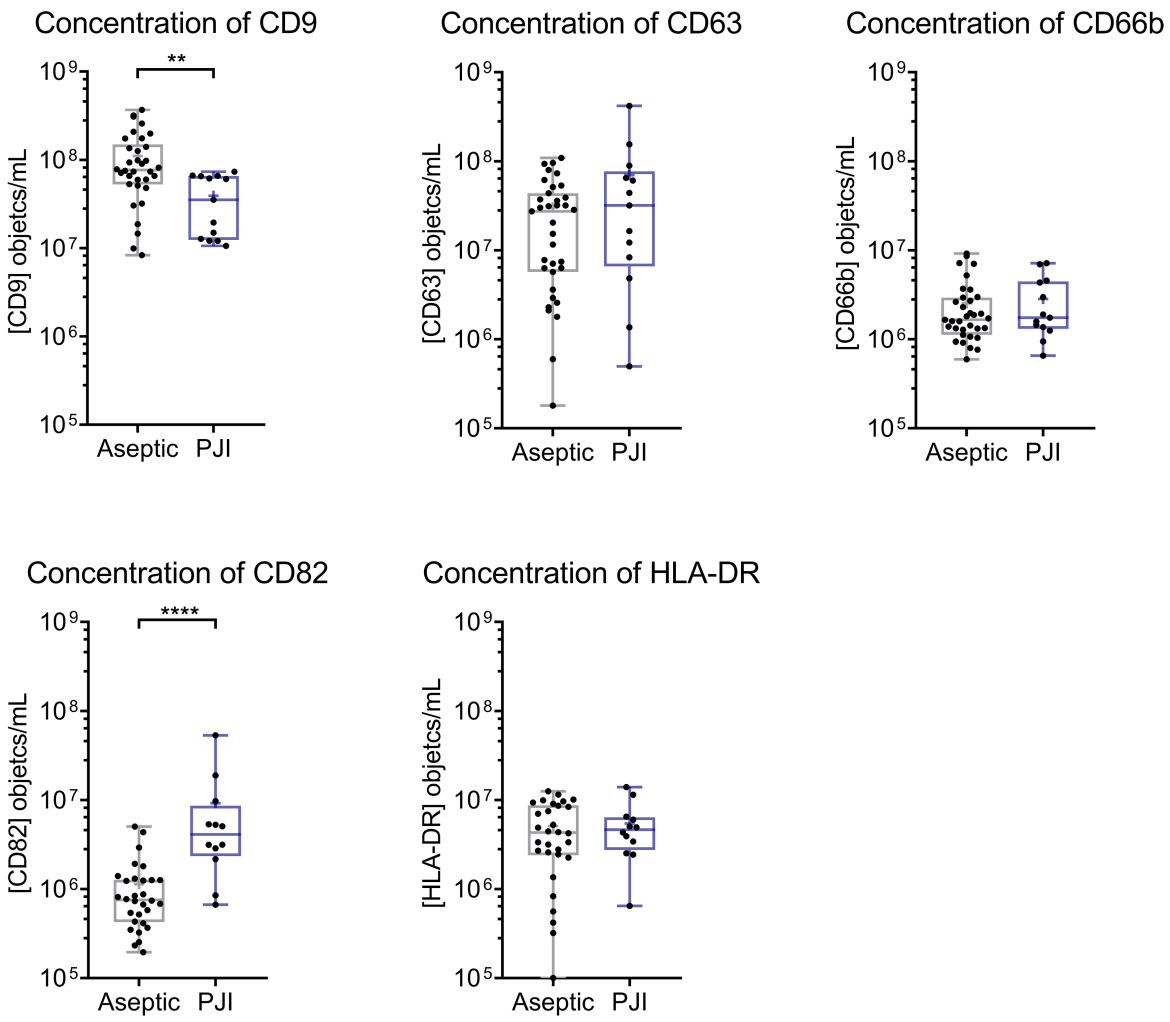
Synovial fluids of patients with aseptic endoprosthesis loosening contain significantly more CD9^+^ and significantly fewer CD82^+^ EVs than those of PJI patients. Aliquots of marginally preprocessed synovial fluids were individually labeled with anti-CD9, anti-CD63, anti-CD66b, anti-CD82, and anti-HLA-DR antibodies and analyzed by IFCM as depicted in Figure 1. The concentration of recorded EVs is provided as objects/mL synovial fluid. Data are presented as dot plots with median and range. Significances were determined using the Mann-Whitney test. Drawing Software: GraphPad. EV: Extracellular vesicle; PJI: periprosthetic joint infection; IFCM: imaging flow cytometry. ^**^P < 0.01; ^****^P < 0.0001.

Applying the same analysis strategy as before, aliquots of the marginally preprocessed synovial samples of the 35 patients with aseptic joint effusion and of the 13 PJI patients were labeled individually with each of the five selected antibodies and analyzed exactly as described before [Figure 1A]. The results revealed that CD9^+^, CD63^+^, and CD66b^+^ EVs were present in all samples. Due to technical limitations, CD82^+^ and HLA-DR^+^ EVs could not be detected in one PJI sample, otherwise such EVs were detected in all other samples. These values refer to the concentrations of EV subpopulations positive for the respective surface markers. The concentration of marker-positive EVs varied from 10^5^ to 10^9^ positive EVs per mL of given synovial fluids [Figure 3]. The Mann-Whitney U test was employed to identify statistically significant differences in the EV concentration of the two cohorts. The analysis revealed differences in the amounts of CD9^+^ and CD82^+^ EVs in the synovial fluids of both patient cohorts. While synovial fluids of patients with aseptic joint effusion contained significantly more CD9^+^ EVs (median: 7.7 × 10^7^, range: 8.3 × 10^6^ to 3.7 × 10^8^, *n* = 34, *P* < 0.01) than those of PJI patients (median 3.5 × 10^7^, range: 1.2 × 10^7^ to 7.3 × 10^7^, *n* = 13), their CD82^+^ EV content was significantly lower (median 7.6 × 10^5^, range: 2.0 × 10^5^ to 5.0 × 10^6^, *n* = 30, *P* < 0.001) than that in the synovial fluid of PJI patients (median 4.1 × 10^6^, range: 6.7 × 10^5^ to 5.3 × 10^7^, *n* = 12). In contrast, no significant differences were observed for CD63^+^ (*P* = 0.383), CD66b^+^ (*P* = 0.647), and HLA-DR^+^ (*P* = 0.711) EVs (Figure 3, all numbers can be found in Supplementary Table 5).

### Diagnostic potential of the abundance of CD9^+^ and CD82^+^ EVs in synovial fluids to identify aseptic and PJI patients

To evaluate the diagnostic potential of EVs within synovial fluids for the discrimination of aseptic endoprosthesis loosening and PJI patients, ROC analysis was performed for all recorded EV populations [Supplementary Table 5].

Of all the EV markers analyzed, the abundance of CD9 and CD82^+^ EVs proved to be the most promising candidates for detecting a septic situation.

**Figure 4:**
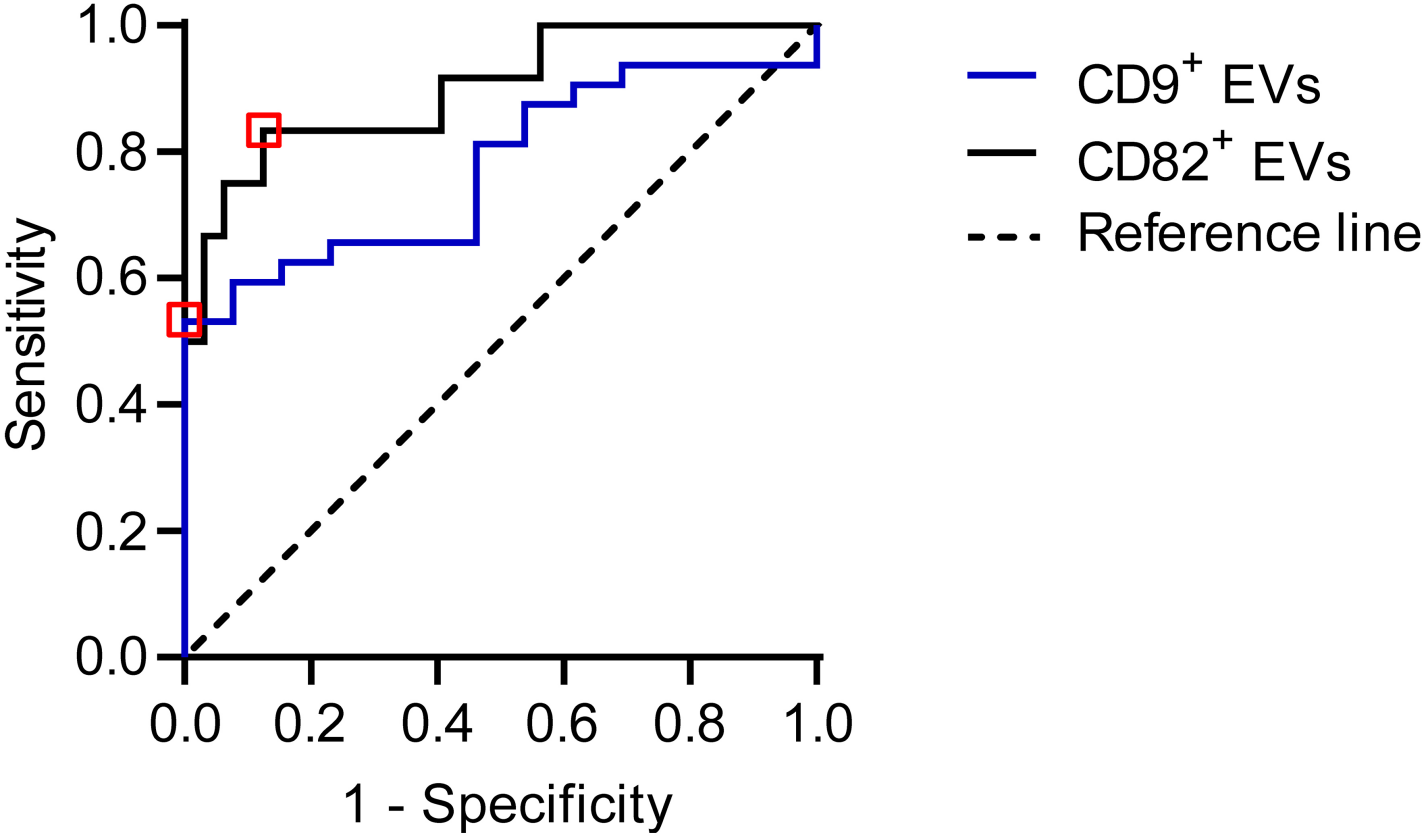
ROC analyses substantiate the diagnostic power of the CD9^+^ and CD82^+^ EV concentration measurements within synovial liquids for the discrimination of aseptic endoprosthesis loosening and PJI. ROC curves of the individual CD9 and CD82 EV concentration values in comparison to the reference line. Red squares show the results of the ROC analysis. Drawing Software: GraphPad. EV: Extracellular vesicle; PJI: periprosthetic joint infection; ROC: receiver operating characteristic.

For CD9, the ROC analysis provided an area under the curve (AUC) value of 0.793 (95%CI: 0.661-0.918) and a threshold level of 7.4 × 10^7^ objects/mL and significantly (*P* = 0.002) differentiates among the aseptic endoprosthesis loosening and PJI samples. Thus, in patients whose abundance of CD9^+^ EVs is above this threshold, aseptic loosening of the endoprosthesis can be predicted with a sensitivity of 58.8%, a specificity of 100, a total misclassification rate of 20.6%, and a false negative rate of 41.2%, indicating that a substantial number of PJI cases remained undetected using CD9+ EVs alone. [Figure 4 and Supplementary Table 5].

According to the ROC analysis, the abundance of CD82^+^ EVs above 2.0 × 10^6^ objects/ml were significantly associated with PJI with a total misclassification rate of 13.3% and a false negative rate of 16.6% or a false positive rate of 10%, respectively (sensitivity: 83.3%, specificity: 90.0%, AUC: 0.886, 95%CI of AUC: 0.764; 1.000; *P* = 0.0001). This observation clearly emphasizes CD82^+^ EVs as robust biomarkers in synovial fluids for the diagnosis of PJI. For the abundances of CD63^+^, CD66b^+^, and HLA-DR^+^ EVs, no significant cut-off level was defined by the ROC analysis.

### CD82^+^ EVs as independent predictors for PJI

According to the defined cut-off values obtained by ROC analysis, the abundance of CD9^+^ and CD82^+^ EVs within synovial fluids was translated into dichotomous variables and included as covariates in binomial logistic regression analysis. Entering these two variables into the regression model, it became evident that only the abundance of CD82^+^ EVs was significantly predictive for aseptic or PJI conditions (Odds ratio: 20.0 and 95%CI: 2.7-144.3, *P* = 0.003).

## DISCUSSION

EVs are increasingly recognized as promising biomarkers, as they reflect the physiological and pathological states of their cells of origin^[36,37]^. Unlike traditional biomarkers such as soluble proteins or cells, EVs can carry complex molecular cargo (proteins, RNAs, lipids) within a protected membrane, making them particularly robust indicators in inflammatory and infectious settings. Despite this potential, clinical translation has been hampered by the technical complexity of EV isolation and analysis. To address this challenge, we applied IFCM, which enables direct phenotypic analysis of individual EVs in minimally processed synovial fluid. This workflow eliminates the need for time- and labor-intensive isolation steps, reducing the diagnostic turnaround time. While we previously demonstrated IFCM’s diagnostic utility in COVID-19 serum samples^[18]^, we here demonstrate its feasibility for analyzing synovial fluid. By enabling rapid EV-based diagnostics at the point of sample acquisition, this approach introduces a novel diagnostic paradigm that is potentially adaptable to intraoperative decision making in prosthesis-related infections. In contrast to conventional EV analysis workflows that require prior isolation or bulk processing, our approach enables direct quantification of surface marker-positive EV subpopulations at the single-object level in native synovial fluid. This reduces both sample handling complexity and turnaround time, while preserving the physiological composition of the fluid, making it highly suitable for diagnostic applications in a clinical setting.

EVs have previously been detected in synovial fluid across various joint pathologies, including inflammatory and degenerative conditions. Distinct EV profiles with diagnostic potential have been reported in the context of rheumatoid arthritis and osteoarthritis^[38,39]^. Recent studies also suggest a potential role of EVs in PJI, particularly regarding EVs derived from activated immune cells and general EV characteristics in revision arthroplasty cases^[40,41]^. However, these studies required EV isolation and thus depended on larger sample volumes, which are often not obtainable. Furthermore, they lacked detailed surface marker profiling and frequently were not conducted at the single-EV level. In contrast, our approach enables the phenotypic characterization of EV subpopulations directly in native synovial fluid using IFCM, eliminating the need for extensive purification. This methodological advantage facilitates the identification of diagnostically relevant EV signatures (CD9^+^ and CD82^+^), positioning our study as a complementary and translational extension to the current literature.

In our current investigation, IFCM was employed to characterize EV populations in the synovial fluids of orthopedic patients experiencing endoprosthesis loosening, attributable to either aseptic or septic processes. Our findings highlight the distinct differences in concentrations of CD9^+^ and CD82^+^ EVs between patients with aseptic loosening and those with PJIs. Through ROC analyses, we determined the diagnostic sensitivity and specificity for these EV markers. Specifically, CD9^+^ EVs exhibited a sensitivity of 58.8% and a specificity of 100%, with a total misclassification rate of 20.6%, thereby indicating their efficacy in predicting aseptic loosening. On the other hand, CD82^+^ EVs showed a sensitivity of 83.3% and specificity of 90.0% for identifying PJIs, coupled with a total misclassification rate of 13.3%, highlighting their utility as potent biomarkers for septic conditions. Further reinforcing these findings, the abundance of CD9^+^ and CD82^+^ EVs were transformed into dichotomous variables and incorporated as covariates in binomial logistic regression analysis. This analysis confirmed the significant predictive value of CD82^+^ EV abundance for distinguishing between aseptic and PJI conditions.

Although classical inflammatory markers such as white blood cells (WBC) and CRP were elevated in PJI patients, their discriminatory power was lower than expected, with partial overlap between groups. All PJI cases in this study were chronic infections, and no patients had received antibiotics prior to joint aspiration. These factors are known to limit the sensitivity of systemic and synovial markers in low-grade infections. EV profiling, by contrast, may capture a more specific and localized immune response within the joint, as reflected by the more distinct separation seen for CD9^+^ and CD82^+^ EVs between the groups.

The diagnostic performance observed in our study, namely the high specificity of CD9 and the high sensitivity of CD82, underscores the complementary value of these markers. This suggests that EV-based analysis could significantly enhance diagnostic accuracy in difficult PJI cases, particularly when combined with existing methods that often lack sensitivity in chronic or low-grade infections. By integrating EV profiling into current diagnostic workflows, the reliability and early detection of PJI may be substantially improved.

Originally, existing diagnostic shortcomings are comparable to those reported for conventional microbiological diagnostics, which often fail to detect the infecting organism in up to 22% of PJI cases and show sensitivities as low as 39% in certain settings^[42]^. Notably, EV-based diagnostics are faster and less costly than conventional approaches, which may require up to two weeks to differentiate aseptic from septic arthroplasty. Therefore, the findings of this study once again underscore the potential of IFCM in uncovering novel EV-based biomarkers.

To our knowledge, IFCM is the only currently available EV analysis platform that supports automated sample loading, enabling medium-throughput analysis with minimal manual handling. However, despite our excitement about the results and the high statistical score obtained for the predictivity of CD9^+^ CD82^+^ EVs in septic endoprosthesis loosening, the number of samples analyzed is far too little for qualifying CD9^+^ and CD82^+^ EVs as real and robust biomarkers for the discrimination of septic and aseptic endoprosthesis loosening patients. At the next level, samples of larger and more diverse patient cohorts need to be evaluated^[43]^. To this end, either IFCM or other single EV analysis platforms need to be developed to fulfill all the requirements for a daily routine diagnostic device, or methods conventionally used in the daily routine need to be adopted for single EV-based diagnostics. For example, enzyme-linked immunosorbent assay (ELISA)-based assays being performed as sandwich ELISA might meet such requirements^[44]^. Moreover, EV-based diagnostics could pave the way for the development of bedside tests that can be conducted during surgery, enabling the rapid identification of septic conditions. Timely recognition may prevent unnecessary revision surgeries and reduce patient burden from painful synovial fluid aspirations, particularly in total hip arthroplasty^[45]^.

From a mechanistic perspective, the elevated levels of CD82^+^ EVs in PJI may reflect the infiltration and activation of immune cells, such as neutrophils and monocytes, which are known to release EVs with pro-inflammatory and immunomodulatory functions^[46]^. CD9^+^ EVs, often linked to vesicle formation and antigen presentation^[47]^, may originate from other synovial cell types or reflect basal vesicle turnover. Whether these EV subpopulations actively contribute to local inflammation and osteolysis or simply mirror underlying pathophysiological changes remains to be clarified in future functional studies.

Due to sample volume limitations in this pilot study, we were unable to analyze co-expression patterns such as CD9^+^CD82^+^ double-positive EVs. Such analyses may uncover diagnostically relevant EV subpopulations or indicate specific cellular origins. Another limitation is the absence of a healthy control group and a stable prosthesis group. Ethical and logistical constraints prevented synovial fluid collection from individuals without a clinical indication. Future studies should address these gaps by including appropriate control groups and implementing broader marker panels to evaluate the diagnostic relevance of co-expressing EV subpopulations. Altogether, our results not only highlight the feasibility of direct EV profiling in synovial fluid but also open the door for developing rapid, minimally invasive diagnostic tools that could transform perioperative decision making in orthopedic infections.

## DECLARATIONS

### Availability of data and materials

The raw data supporting the findings of this study are available within this Article and its Supplementary Materials. Further data are available from the corresponding author upon reasonable request.

### Copyright

© The Author(s) 2025.
