# Supplementary material for: CD9+ and CD82+ extracellular vesicles in synovial fluid differentiate aseptic from septic endoprosthesis loosening
Source: Extracell Vesicles Circ Nucl Acids. 2025 Jul 10;6(3):336–49. doi: 10.20517/evcna.2025.11 (PMC12544082; doi:10.20517/evcna.2025.11)
Supplement: Supplementary file 1 [file evcna-6-3-336-SupplementaryMaterials.pdf]

Supplementary Materials

CD9+ and CD82+ extracellular vesicles in synovial fluid differentiate aseptic from septic endoprosthesis loosening

Tobias Tertel[1], Vera Rebmann[1], Charlotte Bielefeld[2,3], Marcel Haversath[2], Marcus Jäger[2], Alexander Wegner[2,4], André Busch[2,5], Bernd Giebel[1]

[1]Institute for Transfusion Medicine, University Hospital Essen, University of Duisburg-Essen, Essen 45147, Germany.

[2]Department of Orthopedics and Trauma Surgery, University of Duisburg-Essen, Essen 45147, Germany.

[3]Institute of Pathology Nordhessen, Kassel 34119, Germany.

[4]Department of Trauma surgery, Orthopedics and Hand surgery, Klinikum Wolfsburg, Wolfsburg 38440, Germany.

[5]Katholisches Klinikum Essen Philippus-Stift, Essen 45355, Germany.

Correspondence to: Dr. Andrè Busch, Katholisches Klinikum Essen Philippus-Stift, Hülsmannstr. 17, Essen 45355, Germany. E-mail: andre.busch@sana.de; Prof. Bernd Giebel, Institute for Transfusion Medicine, University Hospital Essen, University of Duisburg-Essen, Virchowstr. 179, Essen 45147, Germany. E-mail: bernd.giebel@uk-essen.de

Supplementary Table 1. MSIS (Musculoskeletal Infection Society) Criteria (2011)

**Major Criteria (at least one of the following)**	**Decision**
Presence of a sinus tract communicating with the prosthesis	Infected
Isolation of a microorganism by culture from at least two separate tissue or synovial fluid samples preserved from the affected prosthetic joint	
**Minor Criteria (at least four of the following)**	**Decision**
Elevated serum erythrocyte sedimentation rate (ESR) and serum C-reactive protein (CRP) concentration	Infected
Elevated synovial leukocyte count	
Elevated synovial neutrophil percentage (PMN%)	
Presence of purulence in the affected joint	
Isolation of a pathogen in one culture of periprosthetic tissue or synovial fluid samples obtained from the affected joint	
More than five neutrophils per high-power field (HPF) in five HPF observed from histologic analysis of periprosthetic tissue at 400-fold magnification	

Supplementary Table 2. Patient data whose synovial fluid was analyzed in the current study

	**Aseptic (*n* = 35)**	**PJI (*n* = 13)**
**Female/male**	22/13	7/6
**Age (y)**	of 72.1 ± 12.9 (45-88)	69 ± 15.6 (35-89)
**BMI (kg/m²)**	30.0 ± 7.5 (21.4-52.0)	32.4 ± 9.3 (20.2-55.9)
**Knee/hip/shoulder**	19/15/1	7/5/1
**Multimorbid (> 2 chronic illnesses)**	16	8
**White blood cell count /µL**	7,230 ± 1,650 (3,580-10,820)	8,020 ± 2,170 (4,390-12,920)
**CRP (mg/dl blood)**	1,68 ± 0,65 (0,7-2,4)	8,3 ± 7,04 (0,8-26,7)
**Synovial leukocyte esterase reaction (-/trace/+/++/+++)**	22/10/3/0/0	3/4/3/2/1
**Positive microbiological joint fluid culture**	0	9
Staph. epidermidis	0	3
Staph. epidermidis + Leuconostoc spp.	0	1
Staph. epidermidis + Cutibacterium acnes	0	1
Staph. aureus	0	1
Staph. warneri	0	1
Klebsiella pneumoniae	0	1
Serratia marcescens	0	1

Data are provided as mean values ± SD.

Supplementary Table 3. Laser settings and detection channels/filters used

**Laser [nm]**	**used Power [mW]**	**max. Power [mW]**	**Filter [nm]**
375	70	70	BV421 (Ch07) 435-505
488	100	100	FITC (Ch02) 480-560
561	200	200	PE (Ch03) 560-595<br>ECD (Ch04) 595-642
648	150	150	APC (Ch11) 642-745
785 (SSC)	70	70	SSC (Ch06) 756-780

Supplementary Table 4. Mean EV numbers and concentration ranges of recorded EV populations in the synovial fluids of patients with aseptic endoprosthesis loosening or in that of PJI patients, respectively

**Potential Biomarker**	**Aseptic joint effusion Median (Range; objects/mL)**	**PJI Median (Range; objects/mL)**	***P*-value**
**CD9**	7.7×10^7 (8.3×10^6-3.7×10^8)	3.5×10^7 (1.2×10^7-7.3×10^7)	< 0.01*
**CD63**	$2.7\ x10^7$ (1.8×10^5-1.1×10^8)	3.2×10^7 (5.0×10^5-4.2×10^8)	0.383
**CD66b**	$1.66x10^6$ (6.0×10^5-9.2×10^6)	1.75×10^6 (6.6×10^5– 7.2×10^6)	0.647
**CD82**	$7.56x10^5$ (2.0×10^5-5.0×10^6)	4.1×10^6 (6.7×10^5-5.3×10^7)	< 0.001*
**HLA-DR**	$4.3x10^6$ (3.0×10^4 -1.3×10^7)	4.6×10^6 (6.5×10^5-1.4×10^7)	0.711

*Indicates statistically significant differences in particle concentrations between both groups calculated by using Mann-Whitney Test.

Supplementary Table 5. Parameters reflecting diagnostic accuracy of EV-associated synovial markers

Parameter	*n*	Cut-off	Sensitivity	Specificity	Misclassification rate	AUC	95%CI of AUC	P^*
**CD9**	47	7.4×10^7	58.8	100	20.6	0.793	[0.660789; 0.918396]	0.002
**CD82**	42	2.0×10^6	83.3	90.0	13.3	0.886	[0.763885; 1.000000]	0.0001
**CD63**	48	4.4×10^7	46.2	77.1	38.4	0.585	[0.381760; 0.787470]	0.372
**CD66b**	48	4.0×10^6	30.8	85.3	41.8	0.545	[0.355336; 0.734774]	0.634
**HLA-DR**	42	3.4×10^6	75.0	46.7	39.2	0.539	[0.348565; 0.729213]	0.697

*Wilcoxon-Mann-Whitney-Test.
